# Polydopamine Nanocomposite Hydrogel for Drug Slow-Release in Bone Defect Repair: A Review of Research Advances

**DOI:** 10.3390/gels11030190

**Published:** 2025-03-08

**Authors:** Xiaoman Li, Jianhua Tang, Weiwei Guo, Xuan Dong, Kaisen Cao, Fushan Tang

**Affiliations:** 1 Department of Clinical Pharmacy, Key Laboratory of Basic Pharmacology of Guizhou Province and School of Pharmacy, Zunyi Medical University, Zunyi 563006, China; manmanoooo@126.com (X.L.); gww4567@outlook.com (W.G.); dongxuan07202000@163.com (X.D.); kscao20250227@163.com (K.C.); 2 Key Laboratory of Basic Pharmacology of Ministry of Education and Joint International Research Laboratory of Ethnomedicine of Ministry of Education, Zunyi Medical University, Zunyi 563006, China; 3 Key Laboratory of Clinical Pharmacy of Zunyi City, Zunyi Medical University, Zunyi 563006, China; 4 Cancer Research UK Manchester Institute, The University of Manchester, Cheshire SK10 4TG, UK; jianhua.tang@manchester.ac.uk

**Keywords:** polydopamine, nanocomposite hydrogel, bone defect repair, drug delivery system

## Abstract

In recent years, hydrogels have emerged as promising candidates for bone defect repair due to their excellent biocompatibility, high porosity, and water-retentive properties. However, conventional hydrogels face significant challenges in clinical translation, including brittleness, low mechanical strength, and poorly controlled drug degradation rates. To address these limitations, as a multifunctional polymer, polydopamine (PDA) has shown great potential in both bone regeneration and drug delivery systems. Its robust adhesive properties, biocompatibility, and responsiveness to photothermal stimulation make it an ideal candidate for enhancing hydrogel performance. Integrating PDA into conventional hydrogels not only improves their mechanical properties but also creates an environment conducive to cell adhesion, proliferation, and differentiation, thereby promoting bone defect repair. Moreover, PDA facilitates controlled drug release, offering a promising approach to optimizing treatment outcomes. This paper first explores the mechanisms through which PDA promotes bone regeneration, laying the foundation for its clinical translation. Additionally, it discusses the application of PDA-based nanocomposite hydrogels as advanced drug delivery systems for bone defect repair, providing valuable insights for both research and clinical translation.

## 1. Introduction

Bone defects due to trauma, infection or osteoporosis pose a substantial challenge to the global healthcare system, affecting over 20 million patients annually [1,2,3]. Currently, autologous and allogeneic bone grafts remain the gold standard for the treatment of bone defect repair, but other repair strategies are necessary due to high donor site morbidity, limited availability, and immune refractory reactions [4,5]. In addition, effective bone repair requires sustained pharmacological activity to promote osteoblast proliferation and bone regeneration, a requirement that synthetic implants and systemic drug therapies often fail to meet due to issues of malintegration, off-target toxicity, and inadequate control over osteogenic factor delivery [6,7,8]. As a result, there is an urgent need to develop a drug delivery system with excellent slow-release properties to enhance bone repair and overcome the limitations of existing treatment options. Combining osteogenic drugs with biocompatible and biodegradable carrier materials can create a system that ensures effective loading and controlled release of drugs, thereby sustaining therapeutic action at the defect site and enhancing efficiency while minimizing systemic side effects [9].

Hydrogel shows unique advantages in bone tissue engineering due to its three-dimensional hydration network and extracellular matrix (ECM)-like microenvironment [10,11,12]. It not only has good biocompatibility but also effectively promotes bone regeneration by supporting cellular infiltration, facilitating nutrient diffusion, and enabling local drug delivery [13,14,15]. However, conventional hydrogels often lack the mechanical elasticity and dynamic responsiveness required for load-bearing applications or controlled therapeutic release [16,17,18]. These challenges have led researchers to work on the development of enhanced hydrogels to optimize their mechanical elasticity and dynamic responsiveness to meet the clinical needs of bone regeneration.

**Figure 1:**
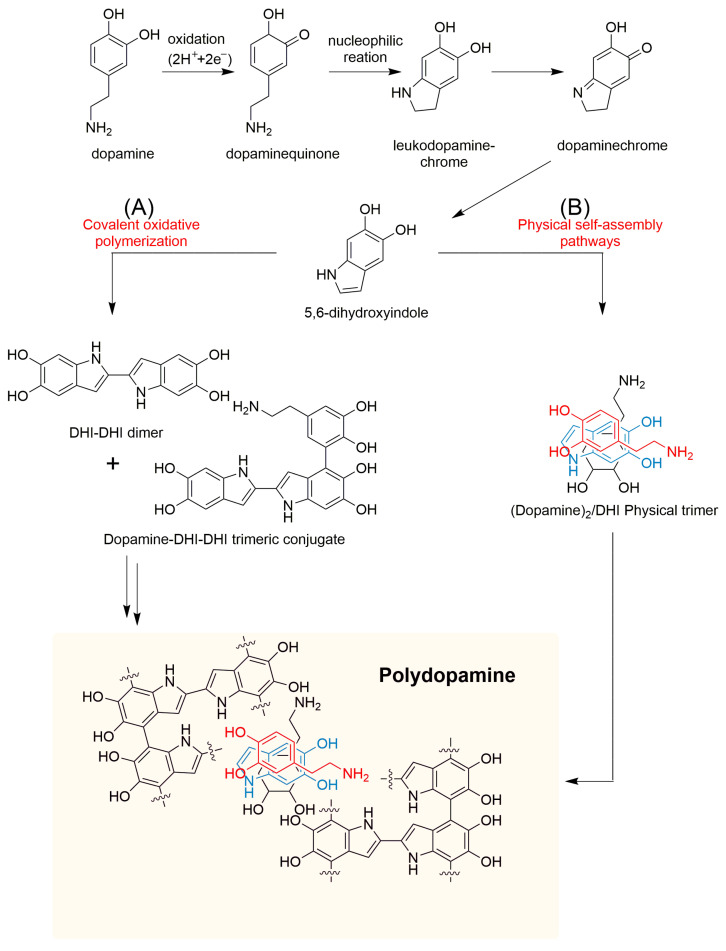
Two synthetic pathways of polydopamine: (A) Covalent polymerization pathway, (B) Non-covalent self-assembly pathway [21].

**Figure 2:**
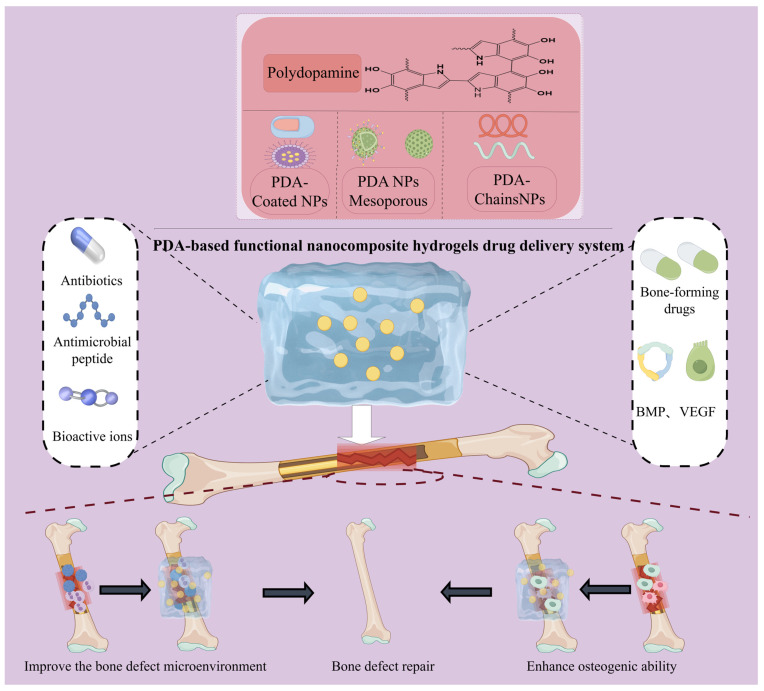
Schematic representation of PDA nanocomposite hydrogel applied to bone defect repair.

Inspired by the adhesive properties of marine mussels, polydopamine (PDA)—a dopamine-based polymer—has emerged as a promising material in the development of advanced hydrogels and bone tissue engineering [19,20]. PDA is formed through the spontaneous polymerization of dopamine monomers under alkaline conditions. Its formation mechanism involves multiple pathways, including both noncovalent self-assembly and covalent polymerization [21] (Figure 1). Initially, dopamine is oxidized to dopamine quinone (DQ), which undergoes cyclization and rearrangement to produce intermediates such as 5,6-dihydroxyindole (DHI) and indole quinone. These intermediates are further polymerized to form oligomers, ultimately producing PDA [21,22]. The resulting polymer is rich in catechol groups, which confer several functional properties, as follows: (i) strong interfacial adhesion, allowing stable binding to both organic (e.g., hydrogels, peptide copolymers) and inorganic (e.g., metal and silicon-based) substrates, facilitating composite material design [23,24,25]; (ii) photo-thermal responsiveness, as the conjugated single-double bonds in its structure enable significant near-infrared (NIR) absorption and efficient photothermal conversion, enabling remote drug release and antimicrobial effects [26,27,28,29]; and (iii) multifunctional modification potential, with abundant amine, imine, and aromatic ring structures that facilitate efficient drug loading and targeted delivery via chemical reactions or π-π stacking [30,31,32,33]. Additionally, PDA’s antioxidant and immunomodulatory properties, such as promoting macrophage M2 polarization, contribute to a pro-regenerative microenvironment for bone repair, enhancing osteogenesis and angiogenesis [34,35]. The diverse nanostructural forms of PDA—such as nanosheets [36], nanoparticles [37], nanocapsules [38], and mesoporous PDAs [39]—offer high modification flexibility, optimizing drug delivery and bone repair effectiveness. Figure 2 illustrates how different PDA structures integrated with hydrogels enable controlled drug release in bone defect repair, regulate inflammation, and promote bone and vascular remodeling. These attributes make PDA a key component in enhancing the mechanical properties and bioactivity of hydrogels, providing a novel approach for developing a smart, responsive platform for bone repair.

Despite significant research on PDA-based nanocomposite hydrogels, current reviews often focus on the physicochemical properties of PDA, with less emphasis on its role in bone repair mechanisms or a systematic classification of PDA hydrogel composites. This paper aims to bridge this gap by delineating the molecular and cellular mechanisms through which PDA promotes bone regeneration, including macrophage polarization, angiogenesis, and Runx2-mediated osteogenesis. It provides a comprehensive classification of PDA-based nanocomposite hydrogels, highlighting design principles, drug loading strategies, and preclinical outcomes. The paper also identifies challenges in clinical translation, such as polymerization variability and long-term biocompatibility, and suggests directions for materials optimization. By integrating materials science with orthopedic application, this study seeks to expedite the development of PDA hydrogels as a versatile platform for bone defect repair. With this comprehensive review, we hope to pave the way for future research and clinical applications of PDA nanocomposite hydrogels in bone defect repair.

## 2. Application and Function of PDA in Bone Defect Repair

A variety of organic and inorganic nanodrug delivery systems have been developed for the treatment of bone diseases, such as silica nanoparticles, gold nanoparticles, calcium phosphate nanoparticles, chitosan nanoparticles, liposome nanoparticles, and polymer nanoparticles [40,41]. However, these systems still face some important limitations, including low drug loading, limited physiological compatibility, poor degradability, and too fast drug release rates [42,43]. To address these drawbacks, PDA has gradually attracted widespread attention as an adjunctive drug delivery system in recent years. PDA not only significantly enhances drug loading capacity, but also improves drug delivery by increasing cell capture efficiency. In addition, PDA itself has a photothermal effect, which can respond to the stimulation of an external light source and thus regulate drug release. In addition, PDA can promote cell adhesion and regulate cell differentiation, which makes it a material with wide application potential in the treatment of orthopedic diseases. Studies have gradually revealed [44,45,46] the multifaceted functions and mechanisms of PDA in bone regeneration, and that it can provide favorable conditions for bone tissue repair by altering the microenvironment, regulating the immune response, and promoting the proliferation and differentiation of osteoblasts.

In this section, we will focus on the different functions of PDA in promoting bone regeneration, especially how it can be further developed in bone defect repair through photothermal effects, cell adhesion enhancement, and regulation of differentiation.

### 2.1. Anti-Inflammation

The inflammatory response plays a crucial role in physiological processes such as tissue injury and bone defect repair. On the one hand, angiogenesis triggered by immune inflammation contributes to the promotion of bone regeneration. Therefore, excessive suppression of early inflammation may interfere with angiogenesis, thereby affecting the healing process of bone defects [47,48]. On the other hand, excessive immune inflammatory response may lead to localized granulomas and fibrous encapsulation, which in turn prevents stable fixation of the implant [49,50]. For this reason, balancing the intensity and duration of the inflammatory response during bone defect repair is crucial for promoting bone healing. The aromatic ring structure and hydrophilic catechol groups on the surface of PDA have antioxidant properties, which can scavenge free radicals in the body and alleviate oxidative stress, thus reducing the inflammatory response triggered by oxidative stress. Through this antioxidant mechanism, PDA helps to improve the local immune environment during bone repair and promotes the osteogenesis process [51,52,53]. In addition, in the local inflammatory region, PDA can reduce the activity of inflammatory cells and the release of inflammatory factors, such as tumor necrosis factor (TNF)-α, interleukin (IL)-1β, and IL-6, through its unique photothermal effect, thus controlling the inflammatory response and preventing the adverse effects of excessive inflammation on bone tissue repair [54]. At the same time, PDA can also promote macrophage M2 type polarization, thereby creating an immune microenvironment conducive to bone repair [48]. Li et al. [55] showed that PDA could inhibit NF-κB expression by increasing i -кB expression in macrophages, thereby reducing the inflammatory response induced by implants.

### 2.2. Promoting Cell Adhesion and Proliferation

PDA can promote cell adhesion through several mechanisms. First, the amine and catechol groups in PDA can strongly chelate with calcium ions (Ca^2+^). This chelation promotes the interaction of blood Ca²⁺ with phosphate ions (PO_4_^3−^), which in turn precipitates and forms a mineralized coating on the PDA surface [52,56]. This mineralization process not only enhances the bioactivity of the material but also provides an ideal surface for osteoblasts and promotes cell attachment and proliferation. Meanwhile, a calcium phosphate crystal is deposited on the surface of PDA, which usually shows a coral like or amorphous nanohemispherical structure [57,58]. This amorphous calcium phosphate is a precursor for the formation of biogenic bone-like apatite (i.e., the inorganic component of bone), which plays an important role in bone repair [59,60]. Studies have shown that, on PDA surfaces containing calcium phosphate, osteoblasts are able to form larger cell clusters. In contrast, on smooth, pure PDA surfaces, osteoblasts show a tendency to diffuse and have difficulty aggregating. This shows that the rough structure of the mineralized coating on the surface of PDA and the formation of the mineralized layer have a facilitating effect on the adhesion and aggregation behavior of osteoblasts [61].

Secondly, PDA, through its surface hydrophilicity and chemical reactivity, can promote the production of a large number of extracellular matrix (ECM) proteins, such as collagen, fibronectin, and vitronectin, by cells. Binding of integrin receptors on cells to these extracellular matrices can activate intracellular signaling pathways (e.g., FAK signaling pathway) to further regulate cellular value addition and differentiation [62,63,64]. In addition to exhibiting changes in cell adhesion capacity, it was found that cells cultured on the PDA surface showed a significant increase in the number of cells in the S phase and a decrease in the number of cells in the sub-G1 phase. This suggests that the PDA-coated material has a promotional effect on cell proliferation [65]. In summary, PDA not only promotes cell adhesion, but also promotes the mineralization process by regulating cell proliferation, promoting osteogenic differentiation, and through interaction with minerals such as calcium phosphate. This provides an ideal support platform for bone defect repair.

### 2.3. Promoting Osteogenesis and Angiogenesis

It has been found that bone tissue is rich in dopaminergic neurons and expresses various DA receptors [34,44]. These receptors, categorized into five types (D1 to D5) [66,67,68], play critical roles in cellular signaling and regulating numerous physiological processes. PDA materials feature dopamine-related reactive groups on their surfaces, enabling interactions with dopamine receptors on cell surfaces, thereby influencing cellular behavior and responses. In bone tissue, the regulation of the extracellular signal regulated kinase (ERK) signaling pathway significantly affects osteoblast behavior. Wang et al. [69] identified D1 as a specific target through which dopamine promotes osteogenic expression by enhancing ERK phosphorylation. This phosphorylation increases the transcriptional activity of RUNX2, a key osteogenic transcription factor that directly drives osteogenic differentiation. The activity of this pathway may also vary depending on the cell type involved. Furthermore, the dopamine D1 receptor (D1R) regulates osteogenic signaling and promotes bone regeneration by activating signaling pathways such as the cyclic adenosine monophosphate (cAMP)/protein kinase A (PKA)/cAMP responsive element binding protein (CREB) pathway [34,70,71]. The coordinated activity of osteoblasts and osteoclasts is crucial during the later stages of bone repair, maintaining the balance necessary for bone tissue health and reconstruction. Osteoblasts facilitate the formation of new bone, while osteoclasts mediate bone resorption, with their interaction ensuring proper bone metabolism. Excessive bone resorption, however, may lead to osteoporosis, potentially compromising bone repair effectiveness. While findings on PDA’s regulation of osteoblast activity remain inconsistent, some studies suggest it may be closely linked to the local degradation concentration of PDA, a relationship that requires clarifying through further investigation [72].

## 3. Advances in the Application of PDA-Hydrogel Based Drug Slow Release System in the Repair of Bone Defects 

**Figure 3:**
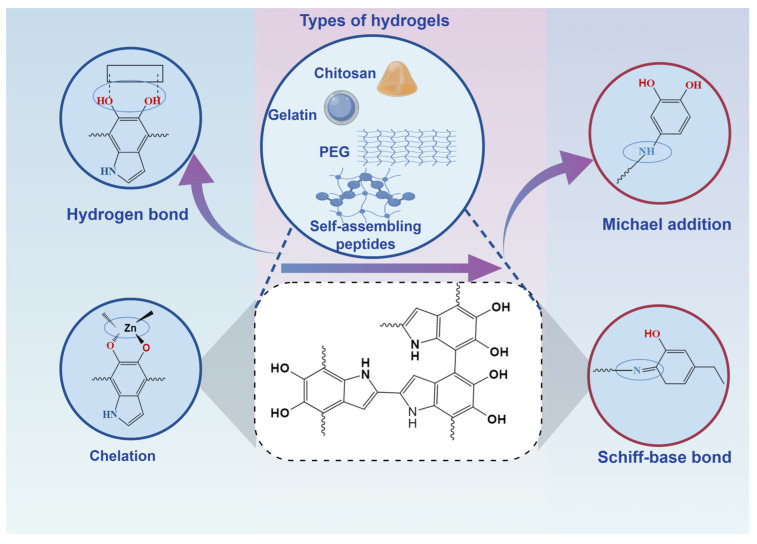
The mechanism of polydopamine-based modified hydrogels.

PDA-based nanocomposite hydrogels, particularly in controlled drug delivery, have driven significant advancements in bone defect repair. These hydrogels are composed of both synthetic and natural polymers. Common synthetic polymers include polyacrylic acid and its derivatives, poly(vinyl alcohol), poly(vinyl oxide), poly(acrylamide), and self-assembling peptides. Natural polymers typically used are collagen, gelatin, hyaluronic acid, alginate, and chitosan. The fabrication of PDA-based hydrogels involves diverse and complex methods, such as hydrogels with dopamine (DA) side-chain branches [73,74,75], hydrogels loaded with PDA nanoparticles (NPs) [76,77,78], and hydrogels incorporating PDA-modified nanomaterials [79,80,81,82]. These hydrogels, including chitosan, gelatin, PEG, and peptides, are often functionalized with PDA to enhance their adhesion properties and bioactivity, offering novel solutions for bone defect repair and laying the groundwork for clinical application (Figure 3). This review explores the unique properties of PDA and examines its integration with various hydrogel types, highlighting several representative composite hydrogels from recent studies (Table 1).

### 3.1. Composite Nanohydrogels of Natural Materials with Polydopamine Nanostructures

Natural polymers are highly favored in bone defect repair due to their superior biocompatibility and biodegradability. However, their hydrogels often lack adequate mechanical properties, limiting clinical applications. Incorporating polydopamine (PDA) into these hydrogels can significantly improve their mechanical attributes and functional capabilities, making them excellent candidates for drug delivery in bone defect repair.

#### 3.1.1. Chitosan-Based PDA Hydrogel

Chitosan, a natural polysaccharide, is widely recognized as an ideal biomaterial for hydrogel preparation due to its excellent biocompatibility, cell adhesion properties, and versatile applications in drug delivery and tissue engineering [134,135]. However, it has limitations, such as poor mechanical strength and instability in high-humidity or acidic environments [136,137]. Incorporating polydopamine (PDA) significantly enhances the functionality of chitosan hydrogels, expanding their potential in biomedical and smart material applications [138,139,140]. These interactions improve the mechanical properties of the hydrogel, increasing stiffness and toughness while reducing swelling and overcoming the mechanical degradation and adhesion loss typically observed in chitosan hydrogels due to their high hygroscopicity [141,142,143]. Additionally, while conventional chitosan hydrogels are often prepared under acidic conditions, their pH stability is limited, and acidic environments can harm bioactive components [144]. Cross-linking with PDA creates a stable network on the chitosan surface, improving water stability and allowing the hydrogel to maintain integrity under acidic or humid conditions, while also enhancing thermal stability [145]. Moreover, PDA-modified chitosan hydrogels offer improved drug delivery capacity by forming a dense network structure that prolongs drug release and enables controlled, slow-release effects [146,147]. The combination of PDA and chitosan not only addresses the limitations of traditional chitosan hydrogels but also advances the development of smart-responsive biomaterials [148]. To further enhance the osteogenic potential, bioactive molecules, mineral particles, or nanomaterials such as hydroxyapatite, silica gel, or calcium–phosphorus complexes can be incorporated.

Rahnama et al. [149] developed an injectable hydrogel combining chitosan, dopamine, and inositol aldehyde for drug delivery. The hydrogel rapidly gels under physiological conditions and exhibits sustained indomethacin release across various pH environments. Its cytocompatibility with L-929 fibroblasts underscores its potential for controlled drug delivery.

**Figure 4:**
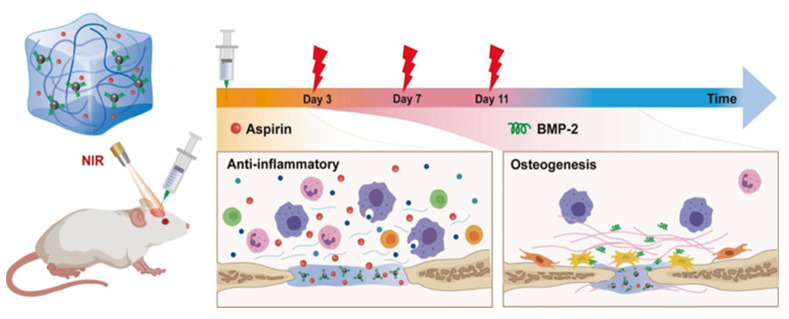
Synthesis of PDA-coated magnesium calcium carbonate microspheres and thermally responsive hydroxybutyl chitosan (HBC) dual responsive hydrogel used for near infrared (NIR) triggered continuous delivery of aspirin (Asp) and BMP-2 to promote in situ calvaria bone regeneration. Adapted with permission from Ref. [82]. Copyright 2022 Carbohydrate polymers.

Wan et al. [82] used PDA-modified hydroxybutyl chitosan hydrogels for bone repair. The hydrogels, with NIR light responsive microspheres, facilitated a sequential release of aspirin and BMP-2, aligning with the bone healing process (Figure 4). This approach offers a novel therapeutic strategy for bone-related conditions.

#### 3.1.2. Alginate-Based PDA Hydrogel

Alginate (Alg), a hydrophilic polysaccharide derived from brown algae, is valued for its excellent biocompatibility and degradability, making it ideal for applications in wound healing, microencapsulation, drug delivery, and 3D scaffolds for bone and soft tissue engineering [150,151]. However, alginate hydrogels often suffer from low mechanical strength and slow degradation rates [152,153,154,155]. Recent studies indicate that incorporating PDA significantly enhances the biocompatibility, mechanical strength, and adhesion of alginate hydrogels, particularly in underwater environments [83,156]. PDA modification improves the mechanical and adhesive properties of the hydrogels through catechol interactions, balancing stiffness and flexibility. This enhancement not only broadens the applicability of alginate hydrogels in tissue engineering but also boosts their diffusion capabilities at contact surfaces [157]. Additionally, PDA-modified alginate hydrogels demonstrate improved cohesion, stability, drug loading capacity, and sustained release properties, offering superior drug delivery performance compared to unmodified alginate hydrogels [158,159]. In summary, alginate–PDA hydrogels provide better control over bioviscosity and osteogenic induction compared to pure alginate hydrogels.

**Figure 5:**
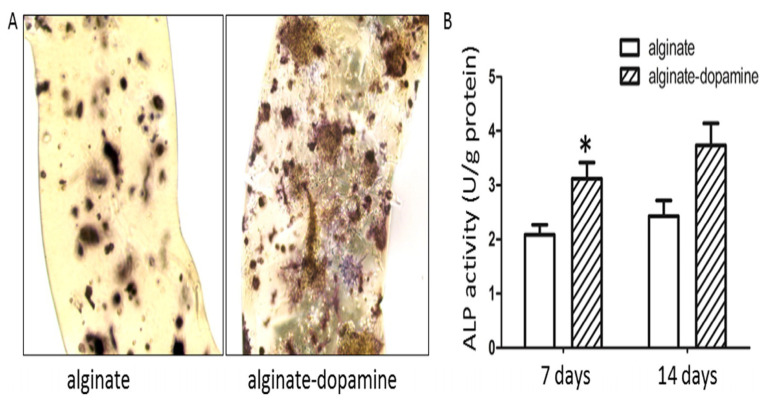
Osteogenic differentiation analysis: (**A**)Alkaline phosphatase (ALP) staining showed the distribution and activity of BMSC in alginate and alginate DA fibrous matrices after 14 days of osteogenic induction. (**B**) ALP activity of BMSC cultured in alginate and alginate dopamine fiber matrices on days 7 and 14 of osteoinduction; data shown as mean ± SD (*n* = 3), “*” indicates significant difference with *p* < 0.05. Adapted with permission from Ref. [83]. Copyright 2016 Materials Science and Engineering: C.

**Figure 6:**
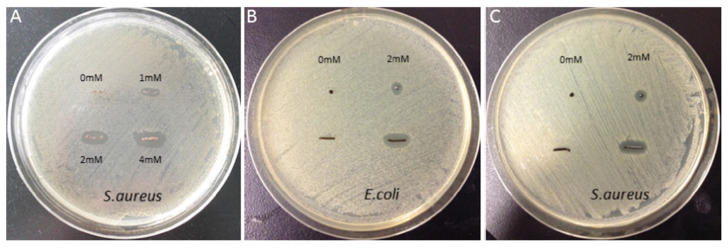
Antibacterial activity analysis: No inhibition zone against * Salmonella * was observed for nanosilver that was not modified with dopamine (**A**). However, alginate dopamine beads and fibers coated with nanosilver exhibited significant inhibition zones against both Gram-positive and Gram-negative bacteria (**B**,**C**). Adapted with permission from Ref. [83]. Copyright 2016 Materials Science and Engineering: C.

Zhang et al. [83] modified alginate with PDA, resulting in hydrogels that promoted MSC cell survival, proliferation, and osteogenic differentiation (Figure 5). Additionally, PDA’s adhesive properties enabled the coating of silver nanoparticles on the alginate PDA gel surface, thereby conferring significant antimicrobial properties to the gel (Figure 6). This hydrogel could be a good tool to promote cell encapsulation and bone regeneration, especially for contaminated bone defects.

#### 3.1.3. Gelatin-Based PDA Hydrogel

Gelatin, derived from the hydrolysis of collagen, is widely used in tissue engineering and drug delivery due to its biocompatibility, degradability, and cell adhesion properties [160,161]. However, gelatin hydrogels face challenges such as weak mechanical strength, poor thermal stability, and limited osteoinductivity, which hinder their effectiveness as bone repair materials [162,163,164]. Similar to chitosan and alginate, the incorporation of PDA enhances gelatin hydrogels by improving their mechanical strength, thermal stability, and cell affinity. These improvements result from noncovalent interactions, particularly those involving the catechol groups of PDA [165,166].

Chen et al. [84] prepared a bifunctional alginate (ALG)/allylated gelatin (GelAGE) hydrogel with UV and Sr^2+^ cross-linking, incorporating PDA particles. This hydrogel showed enhanced mechanical strength compared to other injectable hydrogels. Additionally, the inclusion of PDA@DOX particles increased porosity, facilitating cell penetration. Experimental results revealed that the ALG/GelAGE-PDA@DOX hydrogel achieved a high in vitro killing rate of MG63 cells and exhibited promising bone-like mineralization properties, indicating its potential for bone tissue regeneration.

**Figure 7:**
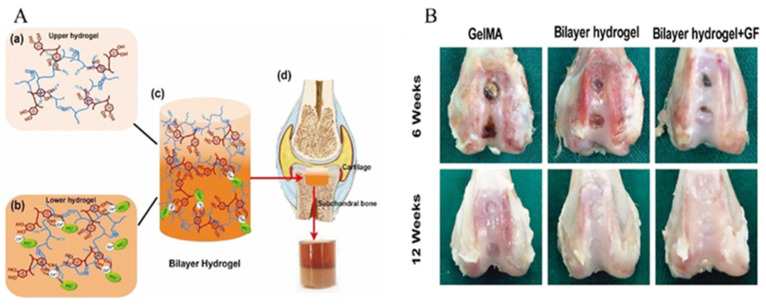
(**A**) Preparation of bilayer hydrogel for cartilage defect repair. (**a**) Gelatin methacryloyl (GelMA) was mixed with polydopamine (PDA) to prepare the upper layer hydrogel for cartilage defect repair. (**b**) Ca^2+^ GelMA was mixed with PO_4_^3−^ GelMA to mineralize hydroxyapatite (HA) in situ, to prepare the lower layer hydrogel for subchondral bone repair. (**c**) Polymerization to generate bilayer hydrogels. (**d**) Schematic of osteochondral defect repair. (**B**) Gross morphological examination of osteochondral defects in the knee joint of rabbits at 6 and 12 weeks after operation was performed. Adapted with permission from Ref. [85]. Copyright 2022 Advanced Healthcare Materials.

Gan et al. [85] developed a mussel inspired bilayer hydrogel for cartilage defect repair (Figure 7). The upper layer comprises a methacrylamide polydopamine (Gel-MA-PDA) hydrogel loaded with transforming growth factor β3 (TGF-β3) to support cartilage regeneration, while the lower layer is a methacrylamide polydopamine/hydroxyapatite gelatin (GelMA-PDA/HA) hydrogel containing bone morphogenetic protein 2 (BMP-2) to aid subchondral bone repair. PDA in the lower layer facilitates hydroxyapatite mineralization, mimicking subchondral bone structure and enhancing mechanical strength. This bilayer hydrogel supports cell proliferation and differentiation, effectively delivering BMP-2 and TGF-β3 for targeted cartilage and subchondral bone regeneration.

### 3.2. Composite Nanohydrogels of Synthetic Materials with Polydopamine Nanostructures

#### 3.2.1. Nanohydrogels Based on Frequently Studied Synthetic Materials

Compared to natural materials, traditional synthetic materials, such as polyethylene glycol (PEG), polyvinyl alcohol (PVA), and poly (methyl methacrylate) (PMMA), are frequently employed to construct strong hydrogels due to their cost effectiveness and robust mechanical attributes [167,168]. Despite these advantages, they typically lack the necessary biocompatibility that facilitates cell adhesion, proliferation, and differentiation. Furthermore, the degradation of polyester compounds like PLGA, PGA, and PLA within the body can result in acidic byproducts that may provoke inflammatory responses, potentially hindering bone healing [169]. The integration of (PDA) into these materials has shown promise in overcoming these limitations, thereby enhancing their utility for bone repair purposes [170,171]. 

Wu et al. [86] have developed a gelatin methacryloyl/poly (methyl methacrylate)/polydopamine (GelMA/PMMA/PDA) photohydrothermal gel aimed at addressing bone defects. This hydrogel underwent rigorous testing through in vitro cell culture experiments and in vivo animal studies to evaluate its biocompatibility and bone repair efficacy. The data revealed its potent osteogenic potential, marking it as a viable treatment for bone defects.

**Figure 8:**
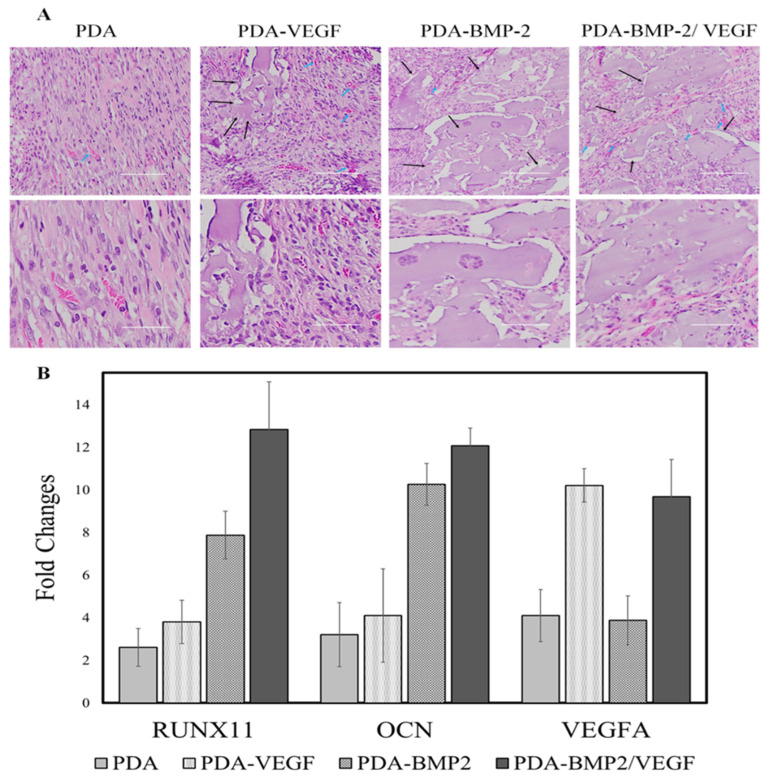
(**A**) H&E staining of decalcified gel matrix sections with cell laden microcarriers: PDA treated (control), VEGF encapsulated, BMP-2 conjugated, and VEGF encapsulated microcarriers with BMP-2 after 6 weeks of subcutaneous implantation. (**B**) Real-time PCR analysis of RUNX2, OCN, and ALP expression after 6 weeks of subcutaneous implantation. Data are presented as means ± standard deviations. Modified from Dashtimoghadam et al. [87]. (2020) under the terms of the Creative Commons Attribution International License (CCBY4.0).

Dashtimoghadam et al. [87] utilized PLGA to create monodisperse microcarriers encapsulating VEGF, which were then functionalized with a biomimetic PDA coating to bind BMP-2. These microcarriers were integrated with an injectable alginate RGD hydrogel to facilitate the controlled release of VEGF and BMP-2 (Figure 8). The findings indicated that the PDA functionalized microcarriers not only improved the immobilization and bioavailability of BMP-2, but also enhanced the attachment and proliferation of mesenchymal stem cells (MSCs), suggesting a promising approach for stem cell therapy in bone defect treatment.

**Figure 9:**
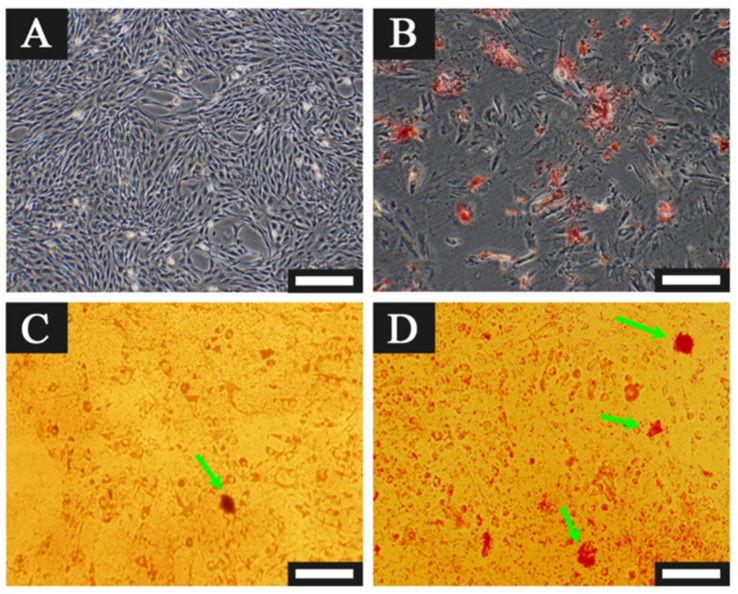
The osteoblastic ability of the AgNPs/PDA gel detected by Alizarin red staining: Alizarin red staining shows osteoblastic activity of AgNPs/PDA gel. (**A**) Cells on a 2D plate. (**B**) Cells on a 2D plate with osteogenic inducer. (**C**) Cells on the gel. (**D**) Cells on the gel with osteogenic inducer. Green arrows indicate calcium nodes. Scale bar: 250 μm. Adapted with permission from Ref. [105]. Copyright 2018 Materials Science and Engineering: C.

**Figure 10:**
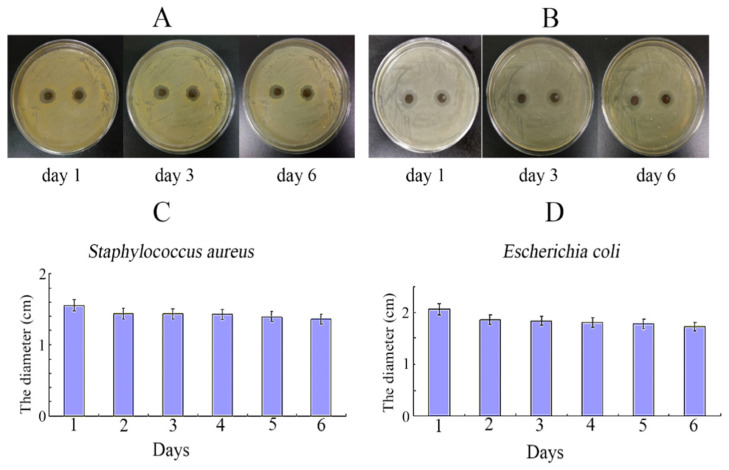
Antibacterial activity of AgNPs/PDA gel: (**A**,**C**) S. aureus groups. (**B**,**D**) *E. coli* groups. (**C**,**D**) Statistical analysis of antibacterial ring diameter. Data are presented as mean ± standard deviation (SD). Adapted with permission from Ref. [105]. Copyright 2018 Materials Science and Engineering: C.

In another study, Xu et al. [105] synthesized a silver nanoparticles/PDA/PEG hydrogel, providing both antimicrobial capabilities and promoting bone mineralization. This utilized PDA mineralization on polyethylene (PEG) hydrogels, and then improved the hydrogel’s adhesion to bone tissue and biocompatibility. Experimental results demonstrated that this hydrogel promotes the proliferation and differentiation of osteoblasts, while inducing bone mineralization and offering antimicrobial protection against infections (Figure 9 and Figure 10). A recent study [27] also describes the preparation of a multifunctional gelatin methacrylate/dopamine gelatin methacrylate adhesive hydrogel coating that includes two-dimensional black phosphorus (BP) nanoparticles coated with dopamine. This multifunctional hydrogel coating, in combination with BP, facilitates photothermal mediated drug delivery and bacterial eradication through photodynamic therapy, aiding in bone healing.

#### 3.2.2. Nanohydrogels Based on Self-Assembling Peptides

Beyond the traditional hydrogels of synthetic materials, peptide-based self-assembling hydrogels have garnered significant attention in materials science and biomedical applications in recent years [172,173]. These peptide-based supramolecular gel scaffolds are extensively utilized in cell culture, tissue engineering, drug delivery, and wound healing, thanks to their straightforward preparation, biocompatibility, and degradability. Previous studies have demonstrated that self-assembling peptide hydrogels are effective in facilitating the repair of bone and cartilage, as well as promoting wound healing [174].

Despite their utility, peptide-based hydrogels often exhibit insufficient functionality and mechanical strength [175,176]. To overcome this limitation, researchers have started to incorporate various inorganic nanomaterials and polymers into these hydrogels, creating hybrid heteropeptide hydrogels that enhance their properties. Studies have demonstrated [177,178,179] that incorporating appropriate amounts of inorganic nanoparticles can significantly improve the mechanical strength and endow new functionalities to the gels. However, achieving compatibility between certain inorganic nanomaterials and the gel matrix can be challenging. The introduction of polymers like (PNIPAAm) can enhance compatibility, but their synthesis is often complex and typically requires organic solvents, which may introduce residues that can adversely affect the biomedical properties of the gels. Therefore, developing novel hydrogel systems that are multifunctionalized and stable in physiological environments is crucial for advancing hydrogel applications in biomedicine.

**Figure 11:**
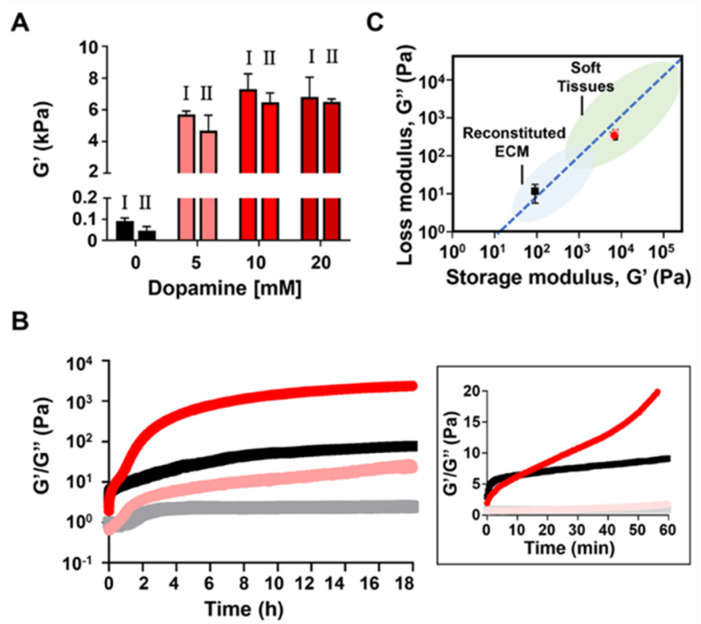
Rheological studies of the 0.5 wt.% MAX1 dopamine gel system. (**A**) Rheological analysis of 0.5 wt.% MAX1 gel 3 days post gelation with varying dopamine concentrations. Left bar (I) shows the initial G’ value before shear thinning (1000% strain, 30 s), and right bar (II) shows the recovered G’ value 10 min after shear thinning. Data are presented as means ± standard deviations. (**B**) Time sweep measurements of 0.5 wt.% MAX1 gel without dopamine (black) and with 5 mM dopamine (red), tracking G and G” (dark and light, respectively) over time at 37 °C, pH 7.4. (**C**) Presheared G and G values of preformed 0.5 wt.% MAX1 gel. Modified from Fichman et al. [180]. (2021) under the terms of the Creative Commons Attribution International License (CCBY4.0).

In light of the mechanical and functional limitations of hydrogels, researchers have started to incorporate polydopamine into peptide hydrogels. This integration not only reduces the critical gel concentration but also significantly enhances their mechanical properties. For example, Fichman et al. [180] showed that self-polymerized dopamine can react with lysine residues in peptides, effectively tuning the viscoelastic properties of the gels (Figure 11). Their findings indicated that incorporating dopamine during gelation significantly increases the storage modulus of the peptide gel while maintaining their shear thinning recovery behavior.

**Figure 12:**
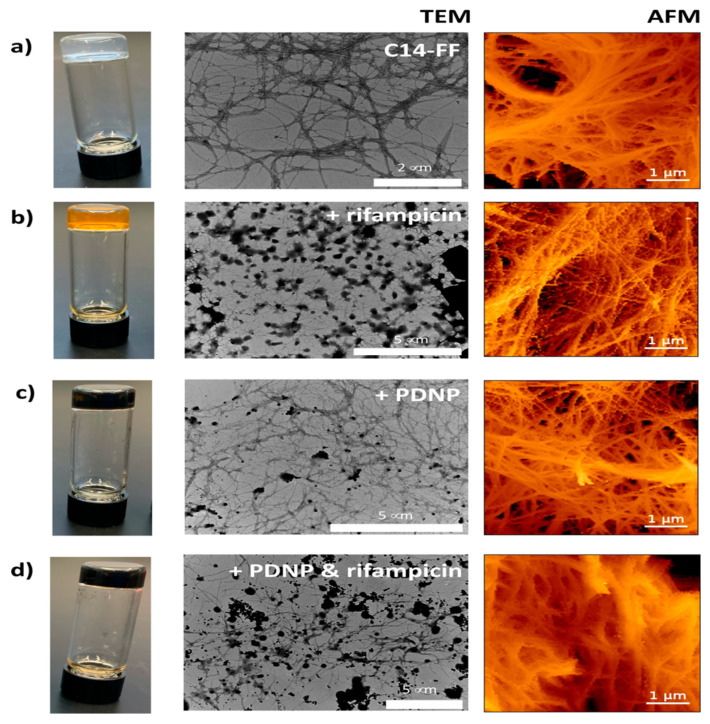
Morphological characterization map of nanocomposite hydrogels: TEM, and AFM images of (**a**) C14-FF gels alone, (**b**) C14-FF + rifampicin, (**c**) C14-FF + PDNP, and (**d**) C14-FF + PDNP + rifampicin. Modified from Falcone et al. [181]. (2021) under the terms of the Creative Commons Attribution International License (CC BY 4.0).

**Figure 13:**
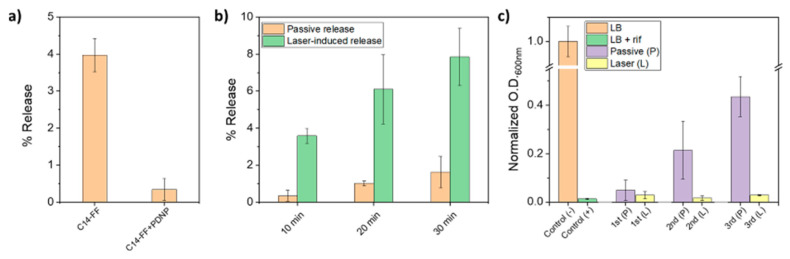
Passive release of rifampicin from the C14-FF hydrogel alone versus the nanocomposite hydrogel with PDNP, and the growth of *Escherichia coli* after laser induced release (**a**–**c**). Data are presented as means ± standard deviations. Modified from Falcone et al. [181]. (2021) under the terms of the Creative Commons Attribution International License (CC BY 4.0).

Additionally, the remarkable photothermal properties of PDA have been utilized to facilitate the loading of drug carrying agents through π-π bonding, leading to the development of a therapeutic platform that integrates photothermal therapy with controlled drug release, thus achieving multifunctionality in hydrogels. For instance, Falcone et al. [181] encapsulated polydopamine nanoparticles (PDNPs) loaded with the hydrophobic drug rifampicin within a C14-FF hydrogel and used laser heating to trigger drug release (Figure 12). By adjusting the laser power and duration, precise control over the drug release rate was achieved. When the laser irradiates the polydopamine layer in the nanocomposite hydrogel, the PDA absorbs the laser energy and converts it into thermal energy, raising the temperature within the hydrogel and altering its structure to facilitate drug release. The results indicate that the C14-FF hydrogel containing PDNPs can sustain the delivery of high concentrations of rifampin after laser induction, effectively preventing bacterial growth (Figure 13). Overall, initiating peptide-based gels in the presence of dopamine offers a simple yet effective method for modulating their viscoelastic mechanical properties, with promising applications in medical wound dressings and controlled drug delivery.

While the use of dopamine self-polymerization as a technique for enhancing peptide-based gels has been proposed, there is a scarcity of detailed reports on its specific applications and effects. Moreover, research into the mechanisms of action and optimization conditions for dopamine in peptide-based gels is limited, particularly in terms of evaluating and comparing effects across different peptide systems and application scenarios. Therefore, despite its potential, the application prospects and advantages of dopamine self-polymerization in peptide-based gels require further validation through more in-depth experimental studies.

## 4. Conclusions and Outlook

Polydopamine (PDA)-based nanocomposite hydrogels represent a promising advancement in drug delivery, offering significant improvements over conventional hydrogels in terms of biocompatibility, drug loading capacity, mechanical strength, and controlled release capabilities. These hydrogels have diverse applications in medicine, including tissue adhesion, sealing, and hemostasis during surgical procedures. By leveraging the unique properties of PDA alongside the advantages of hydrogels, these systems can be engineered to deliver drugs in a controlled manner, often through the integration of nanoparticles or drug carriers. This approach can enhance drug stability, bioactivity, and concentration at target sites, potentially benefiting bone tissue regeneration and addressing bone defect-related conditions.

However, PDA-based hydrogels remain in the research and development stage and have not yet reached widespread commercialization. In contrast, other biomaterials such as chitosan, PEG hydrogel, and fibrin have achieved industrial-scale production, driven by advancements in synthesis, clinical trials, and regulatory approval. For PDA-based hydrogels to follow a similar path, further optimization of the synthesis process and biodegradation properties is essential to ensure consistent polymerization and batch stability, meeting the stringent regulatory standards for medical devices.

Despite their potential, PDA-based hydrogels face three main challenges: (1) limited understanding of PDA polymerization mechanisms, (2) risk of drug leakage during nanoparticle coating, and (3) dependence on cytotoxic cross-linking agents in fabrication. Thus, developing a simplified, one-step method to prepare PDA-based hydrogels without cross-linkers or oxidizers under mild conditions remains a key challenge.

Future research will focus on refining the design and synthesis of PDA-based nanocomposite hydrogels to improve drug-loading efficiency, controlled release, and long-term stability. Collaboration with medical device companies will be crucial to expedite the registration and commercialization of PDA-based products, ultimately advancing these hydrogels into more effective and practical clinical drug delivery tools.

## Figures and Tables

**Table 1:** Recent advances in PDA-based hydrogel delivery system for bone defect repair.

Type of Composite Hydrogel	Types of Cells/Bacteria	Type of Animal Model	Drugs/Growth Factors/Others	Advantages	Disadvantages	Reference
PDA/Hydroxybutyl chitosan hydrogel	hBMMSCs	SD rats	Aspirin	Dual responsive properties	Complex processing	Wan et al. [82]
PDA/alginate hydrogel	BMSCs	/	Silver Nanoparticles	Antibacterial/anti-infective properties	Insufficient mechanical strength	Zhang et al. [83]
PDA/Alginate-allylated double-cross-linked hydrogels	MG63 rBMSCs	/	DOX	Fast kinetic response/High UV cross-linking conversion efficiency/Excellent mechanical properties	Insufficient stability of drug release	Chen et al. [84]
GelMA-PDA/HA hydrogel	BMSCs	New Zealand white rabbits	BMP-2 TGF-β3	Excellent osteochondral repair	Reducing degradation speed	Gan et al. [85]
GelMA/PMMA/PDA hydrogel	BMSCs	BALB/c rats	/	Good biocompatibility/degradation properties	Local overheating	Wu et al. [86]
PLGA-PDA-/Alginate-RGD hydrogel	BMSCs HUVECs HELA MC3T3-E1	Fischer 344 rats SD rats	BMP-2 VEGF BP Doxorubicin Hydrochloride	Precise and timely drug release	Complex processing	Dashtimoghaamet al. [87]
Polyvinyl alcohol (PVA)/PDA@HAP hydrogel	BMSCs	SD rats	Silver NanoparticlesHAP	Long-lasting antimicrobial effect	Induction of cytotoxicity by silver nanoparticles	Li et al. [88]
PDA/Hyaluronic acid methacrylate hydrogel MPs	BMSCs	SD rats Rabbits	Barium titanate nanoparticles Stem cell recruitment peptides	Precise electrical stimulation	Poor stability	Han et al. [89]
PDA hydrogel	hBMSCs	SD rats	CNT PLA	High mechanical strength	Large manufacturing scale	Sun et al. [90]
PDA@E2/HA/GelMA/Gel hydrogel	BMSCs	/	Employing estradiol BMSCs	Adaptation of complex defects	Degradation rate/trigger conditions need to be optimized	Chen et al. [91]
MSN@pDA/Chitosan (CS) hydrogel	SMSCs	SD rats Rabbits	TGF-β3 IGF-I PDGF-BB	Activation of endogenous cartilage repair	Insufficient stability	Li et al. [92]
PDA/Gelatin methacrylate/sodium alginate methacrylate (GA) hybrid hydrogel	HUVECs MC3T3-E1	SD rats	DFO PO_4_^3−^	Improving the microenvironment of bone regeneration	Insufficient biocompatibility/photothermal efficiency	Wu et al. [93]
PDA/Hydroxypropyl chitosan/Gelatin (HG) hydrogel	HUVECs MC3T3-E1	BALB/c rats	aFGF Ti3C2Tx Mxene nanosheets	Accelerating critical bone defect healing	Inadequate biosecurity/limited demand for light control devices	Wu et al. [94]
KGN@PDA/UPy hydrogel	BMSCs	Rabbits	KGN miRNA@CaP NPs	Stable mechanical properties/strong self-healing ability	Complex forming process/insufficient transfection efficiency	Kang et al. [95]
PDA/ PEEK/Gelatin hydrogel	hMSCs	/	BMP-2	Enhanced osteogenic differentiation./Improved bio-inertness of the material	Limited drug loading	Zhang et al. [96]
PDA/Fpolyacrylamide/Silk fibroin hydrogel	BMSCs HUVECs	SD rats	BMP-2DFO	Excellent interfacial adhesion/structural toughness/mechanical stiffness.	Need for multiple photothermal interventions, large implants to trigger foreign body reactions	Li et al. [97]
BML@β-TCP/PDA carboxymethyl chitosan hydrogel	MC3T3-E1	SD rats	BML-284	Multipurpose bone repair	High preparation costs	Wu et al. [98]
GPEGD/PDA hydrogel	MC3T3-E1	SD rats	BMP-2 Heparin	Excellent mechanical properties/biocompatibility	Limited cell infiltration/Insufficient stability of ROS scavengers	Wu et al. [99]
PDA@zeolitic imidazolate framework-8/Soft matrix hydrogel	MC3T3-E1 RAW264.7 HUVECs	SD rats	Zn^2+^	Good structural stability/mechanical support	The complex diabetic microenvironment impairs the therapeutic effect	Wu et al. [100]
PDA/Al/GP/Fibrin hydrogels	EMSCs	SD rats	Al GP	Dual functions of osteoinduction and immunomodulation	Alendronate affects the balance of bone remodeling	Shi et al. [101]
Methacrylated silk fibroin (SFMA)/PDA hydrogel	HUVECs BMSCs	SD rats	MAP	Exhibits good biocompatibility/physicochemical properties	Uneven distribution of photothermal agent/local overheating	Ma et al. [102]
Alginate methacrylate/Alginate/PDA hydrogel	MC3T3-E1	SD rats	Ti_3_C_2_ MXene nanosheets	Good biocompatibility/osteogenic activity/immune-regulatory functions	Phototherapy produces free radicals that damage cells	Wu et al. [103]
PDA/Polysaccharide chitin hydrogel	MC3T3-E1 BMSCs	Wistar male rats	Cu^2+^ Nano HAP	High biocompatibility/Significant osteogenic activity.	Insufficient interfacial bonding strength	Huang et al. [104]
PDA/Polyethylene hydrogel (PEG)	MC3T3-E1	SD rats	AgNPs	Mineralization/anti-infection dual function	Reducing the elasticity of hydrogel	Xu et al. [105]
PDA/Chitosan/Gelatin hydrogel	HUVECs MC3T3-E1 RAW 264.7	SD rats	Hydroxyapatite BaTiO_3_ NPs	Excellent immunomodulation/angiogenesis/osteogenesis/suitable for combat wound repair	Complex material processing	Wu et al. [106]
PDACS/PCL/Hydrogel	HUVECs WJMSCs	/	HUVECs WJMSCs	Synergized to promote osteogenesis/vascularization	Calcium silicate degradation products affect pH/limited microstructure control	Chen et al. [107]
Oxidized sodium alginate (OSA)/Gelatin (Gel)/PDA-nHA hydrogel	BMSCs	Japanese big-ear white rabbits	nHA	Injectable/easy to operate	Long-term stability of nano-hydroxyapatite (nHA) in vivo is insufficient	Liu et al. [108]
CGH/PDA@HAP hydrogel	BMSCs	SD rats	Gallic acid Hydroxyapatite	Enhanced antibacterial and osteogenic synergy	Generation of acidic degradation products	Pang et al. [109]
Characterization of the fucoidan/PDA hydrogel	PDLSCs	/	/	Enhanced osteogenic potential	Quality control of fucoidan sulfate was low	Kwack et al. [110]
Polyacrylamide/PDA hydrogel	MG-63	/	/	Matrix stiffness targets osteosarcoma cell apoptosis	Stiffness parameters need to be highly precise/difficult to adjust in clinical application	Deng et al. [111]
Xanthan gum-PDA hydrogel	BMSCs	SD rats	SDF-1α Mg^2+^	Excellent injectability/mechanical properties	High SDF-1α inactivation	Li et al. [28]
OSA-GelDA@ACP/DA/Ag hydrogel	/	/	ACP DA Ag^+^	Composite hydrogel combines tissue adhesion and anti-infection functions	The hydrogel flexibility/bond strength decreased	Zhong et al. [112]
PDA/Chondroitin sulfate hydrogel	rBMSCs	New Zealand rabbits	SDF-1α	Sustained-release SDF-1α	Lack of control over release kinetics	Wu et al. [113]
ALG/GelAGE-PDA@DOX hydrogels	rBMSCs MG 63	/	Sr^2+^ DOX	Synergistic effect of chemotherapy and photothermal therapy (PTT)	Degree of cross-linking affects the stability of drug release	Chen et al. [84]
PDA/GMS/Osteogenic hydrogel	*P. gingivalis*	SD rats	Amino antibacterial nanoparticle Magnetic nanoparticles	Precision antimicrobial therapy	Magnetic field conditioning devices limit clinical applications	Zhou et al. [114]
PDA/Nano-hydroxyapatite (nHAP) hydrogel	MC3T3-E1	/	PEEK Aspirin	Good biocompatibility/compressive strength/modulus	Poor cell adhesion to the inert surface of PEEK	Li et al. [115]
CS/PDA hydrogel	HUVECs	/	DFO	Enhanced bond strength/angiogenic effect	Short half-life of deferoxamine/frequent injections required	Liu et al. [116]
BNP-PEDOT-PSF-AG hydrogel	PDLSCs	SD rats	Bovine serum albumin nanoparticles Hydrogen sulfide	Promoting alveolar bone regeneration/reversing inflammatory microenvironment under diabetic conditions	Difficulty in controlling the release of H_2_S gas	Fang et al. [117]
Alginate/TOCNF/PDA hydrogel	MC3T3-E1	/	TOCNFs PDANPs	High osteogenic activity	Low structural fidelity after printing	Im et al. [118]
GO-PHA-CPs hydrogel	MC3T3-E1	SD rats	CPs	Exhibits excellent injectability/adhesion/antioxidant activity/osteoinductive properties	Limited self-repair capacity/degradation rate mismatch with bone formation rate	Ma et al. [119]
DA-nano-hydroxyapatite hydrogel	4T1 BMSCs	BALB/c mice	DDP	Synergistic photothermal anti-tumor/bone regeneration capabilities	Photothermal agents are potentially toxic	Luo et al. [120]
MCG-HG-PLGA-PD-B hydrogel	ATDC5 MC3T3-E1	/	BMP-7	Promoting Structural Bionicity in Cartilage Regeneration	Insufficient scaffold porosity connectivity	Jung et al. [121]
Gellan gum/PDA hydrogel	MC3T3-E1	/	ALP	Polydopamine enhances the efficiency of mineralization	Increased material brittleness/complex preparation process	Douglas et al. [122]
PF-127/HAMA/M@S (PH/M@S) hydrogel	rBMSCs HUVEC RAW264.7	Mice	M@S NPs	Cost-effective/easy to synthesize/possesses multiple therapeutic capabilities	Nanoparticles prone to leakage	Liu et al. [123]
PDA/LC hydrogel	BMSCs E. coli S. aureus	SD rats	/	Excellent osteogenic activity/angiogenic capacity/antimicrobial effects	Chitosan causes allergic reactions/complex preparation	Li et al. [124]
Gel-PHA hydrogel	MC3T3-E1	SD rats	nHA	Enhanced mechanical and osteogenic properties of gelatin hydrogels	Reduced hydrogel elasticity	Ma et al. [125]
T/DOP-IL 4/CG-RGD hydrogel	BMSCs	/	IL-4 RGD peptide	IL-4 and RGD synergistically regulate the osteoimmune microenvironment	IL-4 short half-life/RGD overexpression	Li et al. [126]
PDA/Gel-PAA hydrogel	BMSCs	New Zealand rabbits	TGF-β3	Enhances the toughness and cell affinity of PAA hydrogels	Catechol oxidative cross-linking is irreversible/affects controllability of degradation	Yan et al. [127]
AD/CS/RSF/EXO hydrogel	BMSCs	SD rats	Exosomes	Excellent mechanical properties/biodegradability/biocompatibility/the ability	Low efficiency in exosome extraction and loading	Zhang et al. [128]
CTP-SA/TiO2@PDA hydrogel	HUVEC BMSCs Staphylococcus aureus Escherichia coli Streptococcus mutans	SD rats	Cu_2_O TiO_2_ NPs	Enhanced antimicrobial activity	Insufficient mechanical properties	Xu et al. [129]
PDA@SiO2-PRF hydrogel	BMSCs	SD rats	PRF	Multi-level regulation of microenvironment	Proteins are prone to degradation/short shelf life	Ren et al. [130]
Chitosan/ Polydopamine/NO-PVA hydrogel	MRSA MC3T3-E1	SD rats	Ti-RP/PCP/RSNO NO	With combined photothermal/immunotherapy	Photothermal effect damages surrounding healthy tissue	Li et al. [131]
PnP-iPRF hydrogel	BMSCs RAW 264.7	Rats	i-PRF	Multiple pathways regulate the microenvironment	Immunogenic risk has not been completely ruled out	Li et al. [132]
SP@MX/GelMA hydrogel	MG-63 MC3T3-E1	Kunming mice	Tobramycin	Significantly enhances the initial adhesion and proliferation of cells	MXene nanosheets trigger inflammation	Yin et al. [133]
Gelatin-Silkfibroin-Oxidized dextran/PLLA-PLGA-PCL/PDA hydrogel	BMSCs	SD rats	Kartogenin P24 peptides	Excellent cell compatibility/Dual-layer scaffolds synergistically repair osteochondral defects	Weak interfacial bonding strength between the two layers	Zheng et al. [134]
SFO-TA-BGNF-PDA hydrogel	MG-63	/	Bioactive glass	Integrated antimicrobial activity/antiosteosarcoma properties/osteoinduction of multiple functions	Aerogel has low mechanical strength and is not suitable for load-bearing applications	Abie et al. [135]

Abbreviations: hBM(M)SCs, human bone marrow mesenchymal stem cells; MG63, human osteosarcoma cells; rBMSCs, rat bone mesenchymal stem cells; BMSCs, bone marrow mesenchymal stem cells; HUVECs, human umbilical vein endothelial cells; HELA, human cervical cancer cell; MC3T3-E1, mouse embryo osteoblast precursor cells; hMSCs, human mesenchymal stem cells; RAW264.7, murine monocyte-macrophage leukemia cells; EMSCs, ecto-mesenchymal stem cells; WJMSCs, human Wharton’s jelly mesenchymal stem cells; PDLSCs, periodontal ligament stem cells; *P. gingivalis*, *Porphyromonas gingivalis*; 4T1, mouse breast cancer cells; ATDC5, mouse chondrocyte; E. coli, Escherichia coli; S. aureus, Staphylococcus aureus; MRSA, Methicillin-resistant staphylococcus aureus; BMP-2, bone morphogenetic protein-2; DOX, doxorubicin; TGF-β3, transforming growth factor-β3; VEGF, vascular endothelial growth factor; BP, black phosphorus; HAP, hydroxyapatite; CNT, carbon nanotube; PLA, polylactic acid; IGF-I, insulin-like growth factors; PDGF-BB, platelet-derived growth factor-BB; PO_4_^3−^, phosphate; aFGF, acid fibro-blast growth factor; KGN, kartogenin; miRNA@CaP NPs, microRNA@calcium phosphate nanoparticles; DFO, deferoxamine; BML-284, wnt agonist 1; Zn^2+^, zinc ion; Al, aluminum; GP, genipin; MAP, magnesium ascorbyl phosphate; Ti_3_C_2_ MXene nanosheets, titanium carbide MXene nanosheets; Cu^2+^, cupric ion; Nano HAP, nano-hydroxyapatite; AgNPs, silver nanoparticles; BaTiO3 NPs, barium titanate nanoparticles; nHA, nanohydroxyapatite; SDF-1α, stromal cell-derived factor-1α; Mg^2+^, magnesium ion; ACP, amorphous calcium phosphate; DA, dopamine; Ag^+^, silver ion; Sr^2+^, strontium ions; PEEK, polyetheretherketone; TOCNFs, tempo-oxidized cellulose nanofibrils; PDANPs, polydopamine nanoparticles; CPs, bioactive cod peptides; DDP, cisplatin; BMP-7, bone morphogenetic protein-7; ALP; alkaline phosphatase; M@SNPs, spermidine-modified mesoporous polydopamine nano-particles; IL-4, interleukin-4; Cu_2_O, cuprous oxide; TiO_2_NPs, titanium dioxide nanoparticles; PRF, platelet-rich fibrin; Ti-RP/PCP/RSNO, titanium implant; NO, nitric oxide; i-PRF, injectable platelet-rich fibrin.

## Data Availability

Not applicable.
